# The Water Treatment in Typhoid Fever

**Published:** 1887-05

**Authors:** 


### EDITORIAL.

IN another portion of this number of THE JOURNAL AND EXAMINER will be found an abstract of a series of articles by Dr. Ernst Brand of Stettin, Germany, upon the treatment of typhoid fever by means of cold baths. This so-called "method of Brand" became very popular, soon after the publication of the author's monograph upon the subject, but it has gradually fallen into disuse, having been displaced by the more convenient and less tiresome antipyretic method. The author reports 19,000 cases which he analyses, and claims that by a thorough and systematic use of his method the common percentage of mortality of from 16 to 20 may be reduced to from 4 to 8, and that even 4 is too high.

The following statement, which he makes, shows the author's faith in his method of treatment: "I do not say that every case of typhoid fever treated with water will recover, but I have said and I repeat, that every case in which the treatment according to the method of Brand is begun before the fifth day, and regularly and thoroughly carried out, will certainly recover."

The standing of the author, as well as the importance of the subject to those interested in internal medicine, render the topic worthy of careful attention.

